# A STAT3 inhibitor ameliorates CNS autoimmunity by restoring Teff:Treg balance

**DOI:** 10.1172/jci.insight.142376

**Published:** 2021-02-22

**Authors:** Saba I. Aqel, Xiaozhi Yang, Emma E. Kraus, Jinhua Song, Marissa F. Farinas, Erin Y. Zhao, Wei Pei, Amy E. Lovett-Racke, Michael K. Racke, Chenglong Li, Yuhong Yang

**Affiliations:** 1 Department of Neurology, Ohio State University (OSU) Wexner Medical Center, Columbus, Ohio, USA.; 2 Division of Medicinal Chemistry, College of Pharmacy, OSU, Columbus, Ohio, USA.; 3 Department of Medicinal Chemistry, University of Florida, Gainesville, Florida, USA.; 4 Neuroscience program, College of Arts and Sciences, OSU, Columbus, Ohio, USA.; 5 Department of Microbial Infection and Immunity, OSU Wexner Medical Center, Columbus, Ohio, USA.; 6 Quest Diagnostics, Secaucus, New Jersey, USA.

**Keywords:** Autoimmunity, Therapeutics, Autoimmune diseases, Demyelinating disorders, Drug therapy

## Abstract

Reestablishing an appropriate balance between T effector cells (Teff) and Tregs is essential for correcting autoimmunity. Multiple sclerosis (MS) is an immune-mediated chronic CNS disease characterized by neuroinflammation, demyelination, and neuronal degeneration, in which the Teff:Treg balance is skewed toward pathogenic Teffs Th1 and Th17 cells. STAT3 is a key regulator of Teff:Treg balance. Using the structure-based design, we have developed a potentially novel small-molecule prodrug LLL12b that specifically inhibits STAT3 and suppresses Th17 differentiation and expansion. Moreover, LLL12b regulates the fate decision between Th17 and Tregs in an inflammatory environment, shifting Th17:Treg balance toward Tregs and favoring the resolution of inflammation. Therapeutic administration of LLL12b after disease onset significantly suppresses disease progression in adoptively transferred, chronic, and relapsing-remitting experimental autoimmune encephalomyelitis. Disease relapses were also significantly suppressed by LLL12b given during the remission phase. Additionally, LLL12b shifts Th17:Treg balance of CD4^+^ T cells from MS patients toward Tregs and increases Teff sensitivity to Treg-mediated suppression. These data suggest that selective inhibition of STAT3 by the small molecule LLL12b recalibrates the effector and regulatory arms of CD4^+^ T responses, representing a potentially clinically translatable therapeutic strategy for MS.

## Graphical abstract

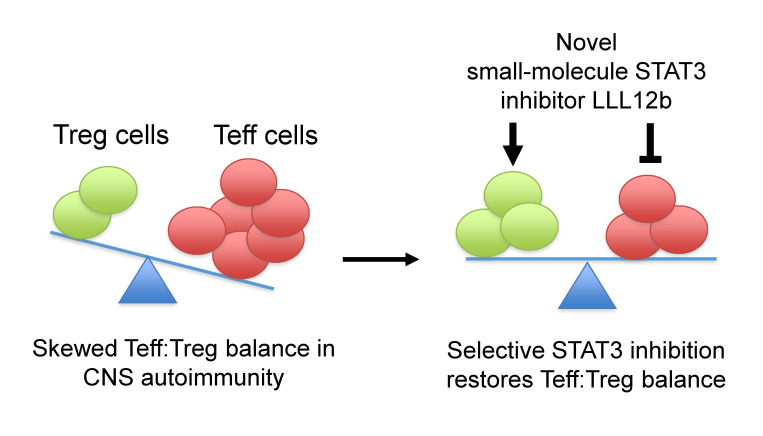

## Introduction

Multiple sclerosis (MS) is an immune-mediated CNS disease characterized by neuroinflammation, demyelination, and neuronal degeneration. MS affects over 1 million people in the United States (1). Current disease-modifying therapies (DMTs) are not effective in all patients, and some of them are associated with significant risks, demonstrating an unmet clinical need for innovative therapies that correct the abnormalities underlying MS pathogenesis. Two subsets of myelin-specific CD4^+^ T effector cells (Teffs), Th1 and Th17 cells, mediate the formation of acute inflammatory lesions and disease progression in experimental autoimmune encephalomyelitis (EAE), a well-defined murine model of MS, and are implicated in MS pathogenesis. CD4^+^ Tregs have the potential to suppress encephalitogenic Teffs and the development of autoimmunity (2). Teff:Treg balance is critical for normal immune function. Teff:Treg balance skewed toward Teffs favors autoimmunity, while therapeutically restoring Teff:Treg balance may lead to resolution of inflammation and amelioration of autoimmunity.

STAT3 is a key regulator of Teff:Treg balance. IL-6, signaling through STAT3, induces the development of highly encephalitogenic Th17 cells (3–5). IL-23, an inflammatory cytokine that is critical for EAE development and progression, signals through STAT3 and expands effector/memory myelin-specific Th17 cells in EAE mice (6, 7). Mice with STAT3 deficiency in CD4^+^ T cells lack naturally occurring Th17 cells and are resistant to EAE induction because of impaired Th17 development (8), suggesting that STAT3 in CD4^+^ T cells is critical for Th17 development and EAE progression. Furthermore, a recent study has shown that STAT3-deficient myeloid cells exhibit impaired antigen-presenting functions and fail to drive the differentiation of encephalitogenic CD4^+^ Teffs (9), suggesting that STAT3 signaling is critical for the antigen-presenting functions of myeloid cells. Therefore, STAT3 inhibition may suppress Th17 development through directly inhibiting STAT3 in CD4^+^ T cells, as well as via inhibiting the antigen presentation of myeloid cells. IL-6^–/–^ and IL-23^–/–^ mice are resistant to EAE induction (8, 10, 11), confirming the critical role of IL-6/IL-23/STAT3 signaling in CNS autoimmunity. In humans, lack of functional STAT3 leads to severely impaired Th17 development, as observed in patients with the hyper-IgE syndrome caused by mutations in *STAT3* gene (12), and contrasts with patients with activating germline mutations in *STAT3*, which cause early-onset multiorgan autoimmune disease (13, 14). Meanwhile, IL-6/STAT3 signaling suppresses Foxp3 expression and completely abrogates the development of inducible Tregs (iTregs) (15, 16). Teffs from patients with autoimmune diseases, including MS, are resistant to Treg-mediated suppression, which is mediated by STAT3 signaling and further skews the functional balance of Teff:Treg toward Teffs (17, 18).

Genome-wide association studies (GWAS) have revealed several *STAT3* variants associated with MS (19). Phosphorylated STAT3 (pSTAT3) was significantly higher in CD4^+^ T cells from relapsing-remitting MS (RRMS) patients in relapse compared with those in remission and healthy controls (HCs) (20). IL-6 level is higher in the cerebrospinal fluid (CSF) of MS patients compared with HCs (21, 22). IL-6 receptor (IL-6R) level is significantly higher in CD4^+^ T cells of MS patients than HCs (18, 23). These suggest that STAT3 inhibition may correct the elevated IL-6/STAT3 signaling and benefit MS. αIL-6 or αIL-6R Ab prevents EAE development when given before disease onset, but it fails to suppress EAE progression when given after EAE onset (24, 25). One of the possible reasons may be that large molecules like Abs have limited CNS penetration. In contrast, small molecules can penetrate the phospholipid membrane of the blood-brain barrier (BBB) by passive or carrier-mediated mechanisms and directly act on Teffs and Tregs in the CNS of patients with established MS. Despite decades of research in developing small molecules specifically targeting STAT3, no selective STAT3 inhibitor has been approved for human treatment to date (26). Using a structure-based drug design strategy, we have developed a selective STAT3 small molecule inhibitor LLL12, which suppresses STAT3 phosphorylation, nuclear translocation, and DNA binding activity (27–29). However, poor pharmacokinetic properties of LLL12 limited its translational potential. LLL12 has poor pharmacokinetic properties with Tmax (the time take to reach maximum concentration) of 5–10 minutes but is cleared in about 30 minutes for p.o. and 60 minutes for i.v. and i.p. administration (Supplemental Figure 1 and Supplemental Table 1; supplemental material available online with this article; https://doi.org/10.1172/jci.insight.142376DS1). Prodrug strategies have shown great success in improving pharmacokinetic properties of small molecules in the past 10 years, with more than 12% of FDA-approved recent small-molecules being prodrugs (30). Thus, we have designed a potentially novel prodrug of LLL12, which restores Teff:Treg balance of CD4^+^ T cells and ameliorates CNS autoimmunity.

## Results

### A potentially novel prodrug of selective STAT3 inhibitor suppresses Th17 cells.

LLL12 is a small molecule that we developed, and it has shown potent and selective inhibition of STAT3 activities in multiple cancer cells in vitro (27–29). We first determined whether STAT3 inhibition with LLL12 suppressed the development of myelin-specific Th17 cells and EAE progression (Supplemental Figure 2 and Supplemental Table 2). LLL12 significantly suppressed STAT3 phosphorylation, IL-17 expression, and the proliferation of myelin-specific CD4^+^ T cells, as well as T cell encephalitogenicity (Supplemental Figure 2, A–H, and Supplemental Table 2). However, preventive or therapeutic administration of LLL12 failed to significantly suppress EAE progression in vivo (Supplemental Figure 2, I–J).

**Figure 1:**
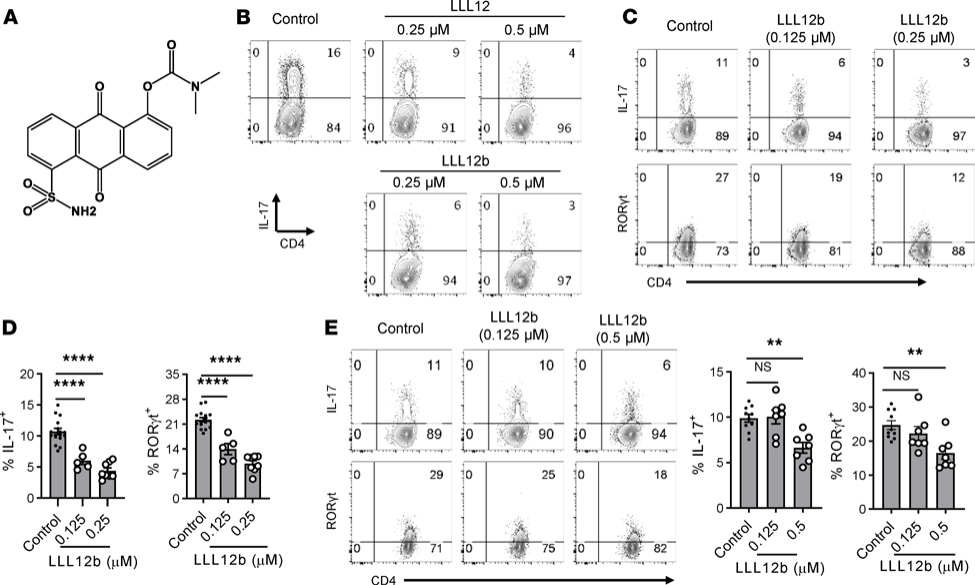
A prodrug of selective STAT3 inhibitor suppresses Th17 development. (**A**) Chemical structure of LLL12b. (**B**) Splenocytes from naive Vα2.3/Vβ8.2 TCR transgenic mice that are specific for MBP Ac1-11 were activated with MBP Ac1-11 plus TGF-β1/IL-6 for 3 days with different concentrations of LLL12b or LLL12. DMSO was used as vehicle control. IL-17 was determined by intracellular staining, gating on CD4^+^CD44^+^ T cells. (**C** and **D**) Purified CD4^+^ T cells were activated with αCD3/CD28 plus TGF-β1/IL-6 for 3 days with different concentrations of LLL12b or vehicle control DMSO. IL-17 and RORγt were determined by intracellular staining, gating on CD44^+^CD4^+^ cells, and compared with 1-way ANOVA (control, *n* = 16; 0.125 μM LLL12b, *n* = 5; 0.25 μM LLL12b, *n* = 8). (**E**) Adherent cells from spleens of WT/B6 mice were cultured with different concentrations of LLL12b or vehicle control DMSO for 4–5 hours. The cells were then cultured with purified CD4^+^ T cells from TCR transgenic 2D2 mice that are specific for MOG 35-55 for 3 days, in the presence of MOG 35-55 and TGF-β/IL-6. IL-17 and RORγt were determined by intracellular staining, gating on CD44^+^CD4^+^ cells, and compared with 1-way ANOVA (control, *n* = 10; 0.125 μM LLL12b, *n* = 7; 0.5 μM LLL12b, *n* = 7). Data are represented as mean ± SEM of 3–5 independent experiments. **P < 0.01; ****P < 0.0001.

**Figure 2:**
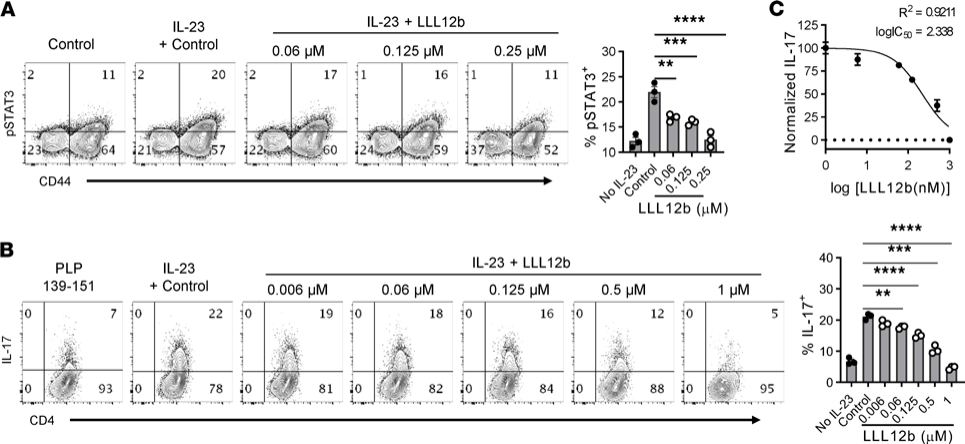
LLL12b suppresses IL-23–induced expansion of Th17 cells from EAE mice. (**A** and **B**) Splenocytes from immunized SJL/J mice were activated with PLP 139-151 plus IL-23 for 30’ (**A**) or 3 days (**B**) with different concentrations of LLL12b. DMSO was used as vehicle control. (**A**) pSTAT3 was determined by phospho flow cytometry, gating on CD4^+^ cells, and compared with 1-way ANOVA (*n* = 3). (**B**) IL-17 was determined by intracellular staining, gating on CD4^+^CD44^+^ T cells. Group means were compared with the control group by 1-way ANOVA (*n* = 3). (**C**) Dose response curve of LLL12b concentration and normalized inhibition of % IL-17^+^CD4^+^ T cells. Data are represented as mean ± SEM of 3 independent experiments. **P < 0.01; ***P < 0.001; ****P < 0.0001.

To improve pharmacokinetic properties of LLL12, 3 prodrugs were designed and synthesized (Supplemental Figure 3). Prodrug LLL12b showed similar suppression of Th17 development as LLL12 and was selected for further investigation. LLL12b is the carbamate prodrug of LLL12 to mainly improve its pharmacokinetic properties (Figure 1A and Supplemental Figures 4 and 5). LLL12b has a sustained effective concentration for i.v. administration of about 4 hours and for p.o. about 8 hours with much better Cmax (the highest concentration of a drug in the blood) and AUC values (Supplemental Figure 6 and Supplemental Table 3), compared with LLL12 (Supplemental Figure 1 and Supplemental Table 1). LLL12b significantly suppressed IL-6–induced IL-17 expression in myelin-specific CD4^+^ T cells from TCR transgenic mice specific for myelin basic protein (MBP) Ac1-11 that were activated with MBP Ac1-11 (Figure 1B and Supplemental Figure 3C). The expression of RORγt, the key Th17 transcription factor, was significantly lower in the LLL12b-treated group compared with the vehicle control (DMSO) group (Supplemental Figure 3D), confirming the suppression of Th17 development by LLL12b. To determine whether LLL12b may suppress Th17 development via inhibiting STAT3 signaling in CD4^+^ T cells, purified CD4^+^ T cells were activated with αCD3/CD28 plus TGF-β/IL-6 in the presence of LLL12b or vehicle control DMSO (Figure 1, C and D). LLL12b significantly suppressed IL-6–induced IL-17 expression in CD4^+^ T cells, at both 0.125 μM and 0.25 μM. The expression of RORγt, the key Th17 transcription factor, was also significantly lower in LLL12b-treated groups (0.125 μM and 0.25 μM) compared with the vehicle control group, confirming that LLL12b has the capacity to suppress Th17 development via inhibiting STAT3 signaling in CD4^+^ T cells. As STAT3 deletion in myeloid cells impairs their capacity for activating myelin-specific T cells (9), we determined whether LLL12b may suppress Th17 development via inhibiting STAT3 signaling in antigen presenting cells (APCs). MOG 35-55–specific CD4^+^ T cells from TCR transgenic 2D2 mice were activated with APCs (adherent macrophages and DCs from spleens of WT/B6 mice) that were pretreated with different concentrations of LLL12b or vehicle control DMSO (Figure 1E). The expression of IL-17 and RORγt was significantly lower in CD4^+^ T cells that were activated with APCs-pretreated with LLL12b (0.5 μM). These data suggest that LLL12b may suppress Th17 development via inhibiting STAT3 signaling in CD4^+^ T cells and APCs. As IL-23/STAT3 signaling promotes the expansion of Th17 cells and is critical for EAE progression, we determined the effects of LLL12b in suppressing IL-23–induced expansion of Th17 cells from EAE mice (Figure 2). IL-23–induced pSTAT3^+^ CD4^+^ T cells and IL-17^+^CD4^+^ T cells were significantly lower in LLL12b-treated groups compared with the control group in a dose-dependent manner (Figure 2, A and B). The dose-response curve of Th17 suppression showed that logIC_50_ of LLL12b is 2.338 nM (*R^2^* = 0.9211) (Figure 2C), similar to that of LLL12 (2.252 nM) (Supplemental Figure 2E). Viability assays showed no significant cellular toxicity on myelin-specific CD4^+^ T cells cultured with different concentrations of LLL12b (Supplemental Figure 7). Together, these data indicate that LLL12b has the capacity to suppress the development and expansion of myelin-specific Th17 cells via inhibition of STAT3 in CD4^+^ T cells and APCs.

### LLL12b regulates the fate decision between Th17 and Tregs.

**Figure 3:**
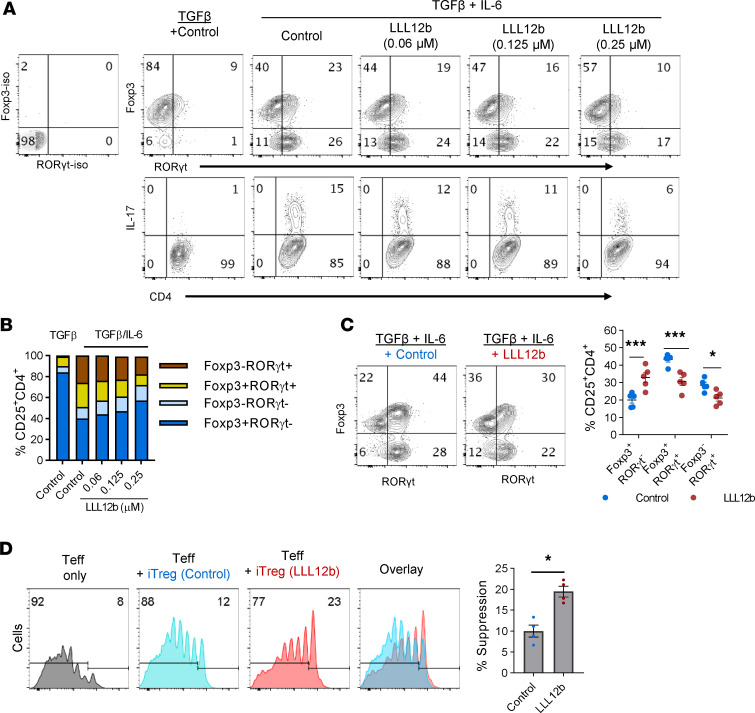
LLL12b regulates the fate decision between Th17 and Tregs. (**A** and **B**) Splenocytes from naive Vα2.3/Vβ8.2 TCR transgenic mice were activated with MBP Ac 1-11 plus TGF-β (4 ng/mL) or TGF-β (4 ng/mL)/IL-6 for 3 days with different concentrations of LLL12b. DMSO was used as vehicle control. RORγt, Foxp3, and IL-17 in CD4^+^ T cells was determined by intracellular staining, gating on CD25^+^CD4^+^ T cells (**A**). (**B**) Stacked bar graph shows subsets of CD25^+^CD4^+^ T cells differentially expressing RORγt and Foxp3 in the groups from the upper panel of **A**. (**C**) CD4^+^ T cells from WT/B6 mice were activated with αCD3/CD28 and TGF-β/IL-6 for 3 days plus LLL12b (0.25 μM) or vehicle control DMSO. RORγt and Foxp3 and in CD4^+^ T cells was determined by intracellular staining, gating on CD25^+^CD4^+^ T cells. Percentage of Foxp3^+^RORγt^–^, Foxp3^+^RORγt^+^, and Foxp3^–^RORγt^+^ cells in LLL12b or control-treated groups were compared with 1-way ANOVA (*n* = 5). (**D**) The cells in **C** were then mixed with CFSE-labeled splenocytes from naive TCR transgenic 2D2 mice that are specific for MOG 35-55 at a 1:4 ratio and activated with MOG 35-55 for 5 days. CFSE was determined by flow cytometry, gating on CD4^+^ cells. Percentage of suppression of the proliferation of CFSE-labeled cells were calculated and compared using Mann-Whitney *U* test (*n* = 4). Data are represented as mean ± SEM of 3–5 independent experiments. *P < 0.05; ***P < 0.001.

Suppression of pathogenic Teffs by Foxp3-expressing CD4^+^ Tregs is an important mechanism of immune tolerance. The balance between Teffs and Tregs decides the outcome of inflammation, and restoring this balance by recalibrating both Teffs and Tregs may provide better therapeutic benefits compared with therapeutic approaches targeting only 1 element. The development of Th17 cells and Tregs is mutually exclusive (31, 32). IL-6/STAT3 signaling plays a critical role in regulating the fate decision of CD4^+^ T cells to become Th17 cells or Tregs in an inflammatory environment such as MS lesions. In addition to orchestrating the molecular signaling of Th17 development, STAT3 may also inhibit *Foxp3* transcription via blocking the binding of pSmad3 to a transcriptional enhancer of Foxp3 promoter (33–35), shifting Th17:Treg balance toward Th17 cells and favoring the development of autoimmunity. This unique role of IL-6/STAT3 signaling in regulating the reciprocal development of Th17 cells and Tregs makes it possible to suppress Th17 and promote Tregs simultaneously. Thus, we determined whether LLL12b may regulate the reciprocal development of Th17 and Tregs and shift RORγt:Foxp3 balance toward Foxp3 (Figure 3). TGF-β alone mainly induced the development of Foxp3^+^RORγt^–^ iTregs (Figure 3, A and B). There is a small population expressing both Foxp3 and RORγt (Figure 3, A and B). When TGF-β and IL-6 coexist, the percentage of Foxp3^+^RORγt^–^ iTregs decreased while Foxp3^+^RORγt^+^ cells and Foxp3^–^RORγt^+^ Th17 cells increased, suggesting that IL-6 promotes Th17 development and suppresses iTreg development (Figure 3, A and B). LLL12b treatment led to increased Foxp3^+^RORγt^–^ iTreg population and to decreased Foxp3^+^RORγt^+^ population and Foxp3^–^RORγt^+^ Th17 cells (Figure 3, A and B), suggesting that LLL12b regulates the fate decision of Th17 and iTregs by promoting iTreg development and suppressing Th17 development, shifting RORγt:Foxp3 balance toward Foxp3. Consistent with the RORγt level, IL-17^+^CD4^+^ T cells were lower in LLL12b-treated groups compared with the control group (Figure 3A). These data suggest that, in an inflammatory environment, LLL12b may alter the composition of a mixed CD4^+^ T population by increasing the Treg population and decreasing the Th17 population. Thus, we hypothesize that LLL12b-treated cells may provide better suppression of Teffs due to increased Tregs in this mixed population. CFSE-based suppression assay was performed to address this hypothesis. Purified CD4^+^ T cells were activated with αCD3/CD28 plus TGF-β/IL-6 in the presence of LLL12b or vehicle control DMSO (Figure 3C). The percentage of Foxp3^+^RORγt^–^ iTregs significantly increased, while the percentage of Foxp3^+^RORγt^+^ T cells and Foxp3^–^RORγt^+^ Th17 cells significantly decreased in the LLL12b-treated group, suggesting that LLL12b may regulate the fate decision of Th17 and iTregs independently of STAT3 inhibition in APCs. LLL12b and control-treated cells were then mixed with CFSE-labeled splenocytes from naive TCR transgenic 2D2 mice that are specific for MOG 35-55 and activated with MOG 35-55 (Figure 3D). The suppression efficiency of LLL12b-treated cells was significantly higher than control-treated cells, and this may be caused by increased iTregs in this mixed population. These data illustrate that LLL12b is able to regulate the fate decision between Th17 and iTregs, which shifts Th17:Treg balance toward Tregs and may lead to resolution of inflammation.

### Therapeutic administration of LLL12b suppresses EAE progression in vivo.

**Figure 4:**
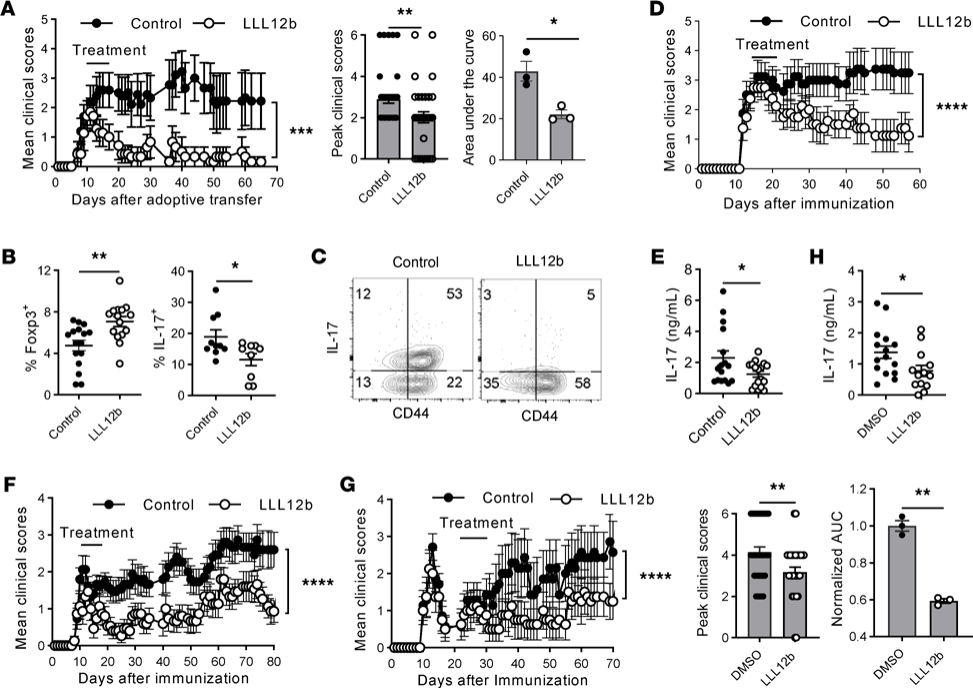
Therapeutic administration of LLL12b significantly suppresses EAE progression. (**A**–**C**) Splenocytes from Vα2.3/Vβ8.2 TCR transgenic mice were activated with MBP Ac1-11 plus IL-6 for 3 days (d) and injected into naive B10.PL mice. LLL12b (10 mg/kg) or vehicle control were injected i.p. into mice daily for 7d starting on d10 after adoptive transfer. Peak clinical scores (control, *n* = 41; LLL12b, *n* = 34) and AUC (*n* = 3) were compared (**A**). On d25 after adoptive transfer, Tregs in the spleens were determined by intracellular staining (control, *n* = 16; LLL12b, *n* = 15) (**B**). Splenocytes (control, *n* = 10; LLL12b, *n* = 9) (**B**) and CNS infiltrating cells (**C**) were activated with MBP Ac1-11 for 3d (**B**) or overnight (**C**). IL-17 was determined by intracellular staining, gating on CD4^+^CD44^+^. (**D** and **E**) C57BL/6 mice were immunized with MOG 35-55 and treated with LLL12b (or control) as described in **A** starting on d14 after immunization. On d29 after immunization, splenocytes were activated with MOG 35-55 for 3d. IL-17 in supernatant was determined by ELISA (control, *n* = 16; LLL12b, *n* = 17) (**E**). (**F**–**H**) SJL/J mice were immunized with PLP 139-151 and treated with LLL12b (or control) as described in **A** starting on d9 (**F**) or d22 (**G** and **H**). Peak clinical scores (*n* = 34) and normalized AUC (*n* = 3) were compared (**G**). On d33 after immunization, splenocytes of mice in **G** were activated with PLP 139-151 for 3d. IL-17 in supernatant was determined by ELISA (LLL12b, *n* = 13; control, *n* = 15). EAE clinical scores were compared with Mann-Whitney *U* test. Bar graphs were compared with unpaired Student’s *t* test. Data represent mean ± SEM of 3–5 independent experiments. *P < 0.05; **P < 0.01; ***P < 0.001; ****P < 0.0001.

Although αIL-6 and αIL-6R Abs showed efficacy in preventing EAE development, they failed to suppress EAE progression when given after EAE onset (24, 25). From a therapeutic point of view, it is critical to determine whether the therapeutic administration of LLL12b after EAE onset suppresses EAE progression. EAE is a well-defined murine model of MS and has been used to study immune mechanisms and evaluate potential therapeutic agents for several decades (36). Many FDA-approved MS treatments were developed, validated, or tested in the EAE model (37–53), indicating that EAE, although with limitations, is still a crucial tool for developing MS therapeutics. The clinical course in different mouse strains resembles specific clinical manifestations of different MS subtypes, so we determined the efficacy of LLL12b treatment in 3 mouse strains (Figure 4). EAE was induced in WT B10.PL mice by adoptive transfer of splenocytes from naive MBP Ac1-11–specific TCR transgenic mice that were activated with MBP Ac1-11 plus IL-6. When 70%–80% of mice developed clinical signs of EAE, the mice were treated with LLL12b (10 mg/kg) or vehicle control daily for 7 days (Figure 4, A–C, and Table 1). The EAE severity, peak clinical scores, and AUC were significantly lower in the LLL12b-treated group compared with the control group (Figure 4A), demonstrating that LLL12b has significant therapeutic benefit in suppressing EAE progression. Tregs (Foxp3^+^CD25^+^CD4^+^) were significantly higher, while Th17 cells were significantly lower, in LLL12b-treated mice (Figure 4, B and C), illustrating a shift of Teff:Treg balance toward Tregs by LLL12b treatment in vivo. This may be due to the inhibition of STAT3 in both CD4^+^ T cells and APCs in LLL12b-treated EAE mice. To confirm this observation and make certain the therapeutic effects are not specific to a certain MHC, antigen, or EAE model, LLL12b (10 mg/kg) or vehicle control was used to treat C57BL/6 mice that were immunized with MOG 35-55 after disease onset (Figure 4, D and E). EAE severity was significantly lower in the LLL12b-treated group (Figure 4D), with decreased IL-17 production in splenocytes from the LLL12b-treated group compared with the control group (Figure 4E), confirming that LLL12b treatment suppresses EAE progression in vivo. Since RRMS is the major subtype of MS that affects more than 85% of MS patients, LLL12b was evaluated in immunized SJL/J mice, which develop a relapsing-remitting disease course resembling RRMS and allows assessment of the treatment efficacy at various stages of disease. The effects of LLL12b were determined when given during the acute (Figure 4F) or remission phase (Figure 4, G and H, and Table 2) in immunized SJL/J mice. EAE severity in the group treated with LLL12b during the acute phase was significantly lower than the control group (Figure 4F), demonstrating that therapeutic administration of LLL12b significantly suppresses acute EAE in a RR-EAE model of MS. When LLL12b was given during the remission phase (days 25–35 after immunization), EAE relapses, including peak clinical scores and AUC, were significantly lower in the LLL12b-treated group (Figure 4G and Table 2), indicating that therapeutic administration of LLL12b significantly suppresses EAE relapses. IL-17 expression was significantly lower in draining lymph node cells of the LLL12b-treated group compared with the control group (Figure 4H). These data suggest that LLL12b may provide therapeutic benefits in suppressing disease relapses and progression in patients with established MS.

### LLL12b restores Th17:Treg balance of CD4^+^ T cells from MS patients in vitro.

**Figure 5:**
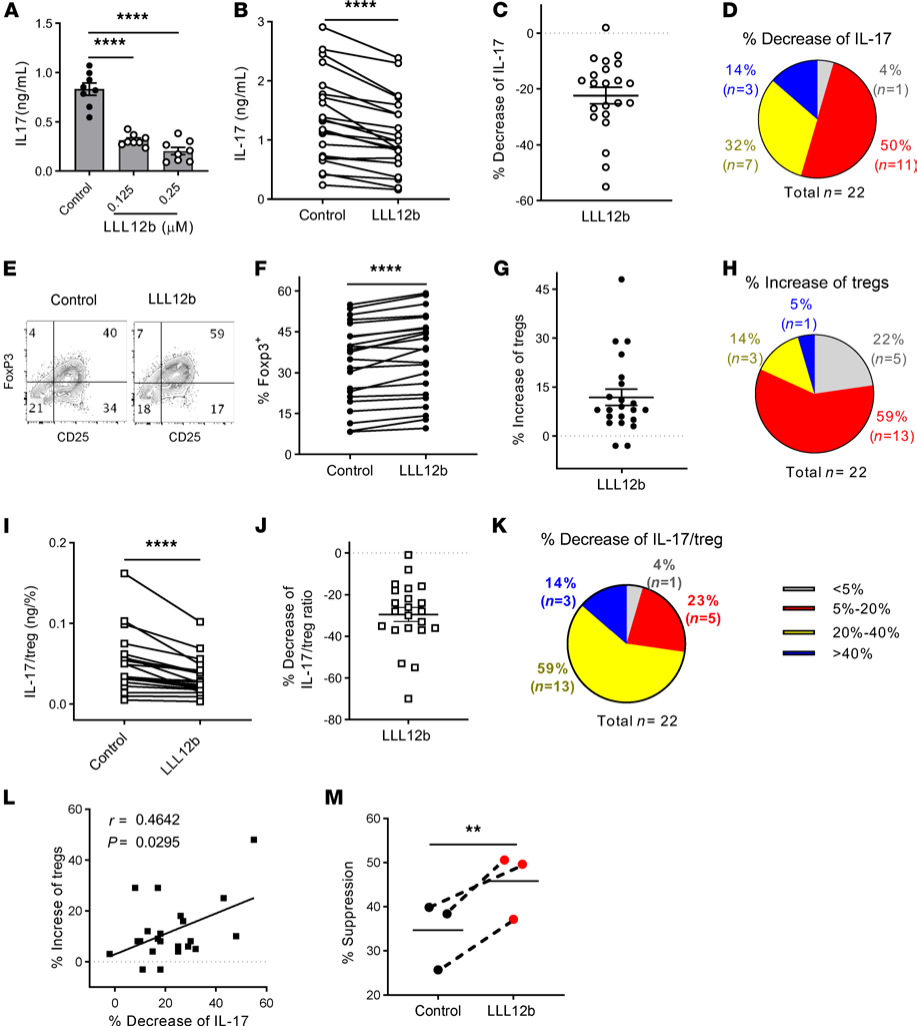
LLL12b restores Th17. Treg balance of CD4^+^ T cells from MS patients. (**A**–**L**) PBMCs from treatment-naive MS patients (*n* = 22) were activated with αCD3/CD28 plus TGF-β/IL-6 for 3 days with LLL12b. DMSO was used as vehicle control. (**A**–**D**) IL-17 in supernatant of 1 patient sample was determined by ELISA and compared with 1-way ANOVA (**A**). IL-17 in LLL12b (0.125 μM) group was compared with control group of the same patient using Wilcoxon matched-pairs signed rank test (**B**). The percentage of decrease of IL-17 was calculated (**C**), and the patients in different ranges were shown in a pie chart (**D**). (**E**–**H**) Tregs (FoxP3^+^CD25^+^) were determined by intracellular staining, gating on CD45RA^+^CD4^+^ cells (**E**), and compared between LLL12b- (0.125 μM) and control-treated groups of the same patient using Wilcoxon matched-pairs signed rank test (**F**). The percentage of increase of Tregs was calculated (**G**), and the patients in different ranges were shown in a pie chart (**H**). (**I**–**K**) IL-17/Treg ratio of each patient was calculated and compared between LLL12b and control groups with Wilcoxon matched-pairs signed rank test (**I**). The percentage of decrease of IL-17/Treg ratio was calculated (**J**) and shown in a pie chart (**K**). (**L**) A nonparametric Pearson correlation test was used to analyze the degree of relatedness between percent increase of Treg and percent decrease of IL-17. (**M**) PBMCs from MS patients (*n* = 3) were activated with αCD3/CD28 under iTreg differentiation condition for 3 days. CFSE-labeled PBMCs from the same 3 patients were cultured with LLL12b (0.25 μM) or vehicle control DMSO for 2 hours, washed and mixed with iTregs cultured cells (Teff:Treg = 16:1), followed by activation with αCD3/CD28 for 5 days. CFSE in CD4^+^ T cells was determined by flow cytometry. The percentage of suppression was calculated and compared with a paired Student’s *t* test. **P < 0.01; ****P < 0.0001.

Previous efforts of targeting total CD4^+^ T cells or CD4^+^ Teffs failed to show significant therapeutic benefit in MS (54–56), suggesting that targeting Teffs alone has a limited benefit for MS. Instead, we need to recalibrate both effector and regulatory arms of CD4 responses to restore peripheral tolerance. Therefore, we determined the effects of LLL12b in regulating Th17:Treg balance of CD4^+^ T cells from MS patients in vitro. PBMCs from treatment-naive MS patients were activated with αCD3/CD28 and TGF-β/IL-6 plus LLL12b or vehicle control DMSO (Figure 5, A–L). LLL12b suppressed IL-6–induced phosphorylation of STAT3 in human CD4^+^ T cells (Supplemental Figure 8). IL-17 production was significantly lower in LLL12b-treated cells compared with control-treated cells (Figure 5, A and B). Decreased IL-17 production (up to 55%) was observed in 21 of 22 LLL12b-treated patient samples (Figure 5, C and D). A moderate decrease (5%–20%) was observed in 50% of patient samples (*n* = 11) (Figure 5D). A higher decrease (20%–40%) was observed in 32% of patient samples (*n* = 7), while a decrease of more than 40% in 14% of patient samples (*n* = 3) was observed. Furthermore, LLL12b significantly enhanced the development of iTregs in CD4^+^ T cells from MS patients (Figure 5, E–H). Increased iTreg development (up to 48%) was observed in 20 of 22 LLL12b-treated patient samples (Figure 5, E–G). A moderate increase (5%–20%) was shown in 59% of patient samples (*n* = 13) (Figure 5H). A higher increase (20%–40%) was observed in 14% of patient samples (*n* = 3), while an increase of more than 40% was observed in 1 patient. To better evaluate the effects of LLL12b on Th17:Treg balance, the IL-17/Treg ratio was calculated and compared between LLL12b- and DMSO-treated groups of each patient sample (Figure 5, I–K). IL-17/Treg ratio was significantly lower in the LLL12b-treated group compared with the control group (Figure 5I). Decreased IL-17/Treg ratio was observed in all 22 patient samples (Figure 5, J and K). A moderate decrease (5%–20%) was observed in 23% of patient samples (*n* = 5) and a higher decrease (20%–40%) was observed in 59% of patient samples (*n* = 13), while a decrease of more than 40% was observed in 14% of patient samples (*n* = 3) (Figure 5K). There was a positive correlation between the percent of decrease of IL-17 and the percent of increase of iTregs (Figure 5L), suggesting that the increase of iTreg development by LLL12b treatment may contribute to the suppression of IL-17 production in CD4^+^ T cells from MS patients. Additionally, CD4^+^ Teffs from MS patients, but not those from HCs, are resistant to Treg-mediated suppression, which is mediated by STAT3 signaling (18). Therefore, we determined whether LLL12b may increase the sensitivity of Teffs from MS patients to the Treg-mediated suppression (Figure 5M). The suppression efficiency of Tregs on LLL12b-treated CD4^+^ Teffs was significantly higher than control-treated CD4^+^ Teffs (Figure 5M), suggesting that LLL12b may correct the defects of Treg resistance of CD4^+^ Teffs from MS patients, which may provide therapeutic benefit for MS patients.

## Discussion

Here, we report that selective targeting of STAT3 with a potentially novel small molecule STAT3 inhibitor has promising translational potential for treating patients with established MS. Therapeutic administration of a small molecule STAT3 inhibitor prodrug LLL12b administered after EAE onset suppressed disease progression in adoptively transferred, chronic, and relapsing-remitting EAE, as well as EAE relapses when given during the remitting phase. We also explored mechanisms underlying the therapeutic effects. We found that LLL12b suppresses IL-6– and IL-23–induced Th17 development and expansion via inhibiting STAT3 in CD4^+^ T cells and APCs. LLL12b regulates the fate decision between Th17 and Tregs in an inflammatory environment, shifting Th17:Treg balance toward Tregs. To evaluate the translational potential of LLL12b, we found that LLL12b shifts Th17:Treg balance of CD4^+^ T cells from MS patients toward Tregs. LLL12b treatment also increases the sensitivity of CD4^+^ T cells from MS patients to Treg-mediated suppression, suggesting that LLL12b has the potential to restore Teff:Treg balance of CD4^+^ T cells in MS patients.

STAT3 signaling plays an important role in suppressing iTreg development and function. Early studies showed that STAT3 is important in the molecular pathway for Foxp3 expression (57, 58). Subsequent studies demonstrated that STAT3 promotes the instability of natural Tregs (nTregs) (59) and may negatively regulate iTreg development by downregulating Foxp3 expression (60). STAT3 may bind to a silencing element of *Foxp*3 locus, which blocks the binding of pSmad3 to enhancer I and suppresses *Foxp3* transcription and iTreg development (33, 34). Moreover, selective inhibition of STAT3 is particularly important in an inflammatory environment such as MS lesions where multiple cytokines are present, including TGF-β and IL-6, since STAT3 is a fate decision point of Th17 and Tregs. Suppression of Th17 development would lead to improved iTreg development because of the reciprocal regulation of Th17 cell and Treg development. To understand the effects of LLL12b in regulating Teff:Treg balance in an inflammatory environment such as MS lesions, we show that LLL12b shifts the balance of RORγt:Foxp3 in CD4^+^ T cells toward Foxp3 (Figure 3), suggesting that STAT3 inhibition with LLL12b regulates the fate decision of Th17 and Tregs. LLL12b may promote iTreg differentiation via inhibiting STAT3 in CD4^+^ T cells activated with αCD3/CD28, as shown in Figure 3C. Although the number of endogenous nTregs is very low, it is possible that LLL12b treatment may alter the plasticity of nTregs, contributing to the increase of Foxp3^+^ Tregs in the LLL12b-treated group. When splenocytes were activated with myelin antigen in Figure 3, A and B, multiple mechanisms may have contributed to this effect. LLL12b may exert its function via inhibiting STAT3 in CD4^+^ T cells and APCs. Since STAT3 signaling is important for the production of inflammatory cytokines in myeloid cells, LLL12b treatment may also alter the cytokine milieu in the microenvironment. To determine whether Tregs in the LLL12b-treated group are functional, we analyzed the suppression of Teffs by the cells in LLL12b- and DMSO-treated groups. Our data show better suppression of Teff proliferation by LLL12b-treated iTreg cultured cells, suggesting that Tregs in the LLL12b-treated group are functional and the increased Treg number may lead to better suppression of Teff proliferation. Further investigation of the suppressive function of sorted Tregs differentiated from naive CD4^+^ T cells of GFP-Foxp3 reporter mice may help to elucidate whether LLL12b treatment increases the suppressive capacity of Tregs. In addition to regulating T cell differentiation, STAT3 has been shown to promote T cell survival and inhibit IL-2 production through upregulation of class O forkhead transcription factors (61). Exogenous IL-2 was added into LLL12b and control-treated groups during Th17 and Treg development, and similar patterns were observed (data not shown) as shown in Figures 1 and 3, suggesting that LLL12b may exert its function in regulating Th17 and Treg development independently of IL-2. Recent studies have demonstrated that iTregs may become unstable and adopt a Teff phenotype under inflammatory conditions (59, 60, 62). iTreg stability contributes significantly to Teff:Treg balance in vivo and is important for the resolution of inflammation. STAT3 has been shown to destabilize Tregs and is required for the conversion of Tregs to Teffs in an inflammatory environment (59), suggesting that STAT3 inhibition may increase iTreg stability. Further investigation is needed to determine the effects of LLL12b in regulating iTreg stability.

From a therapeutic point of review, it is critical to determine the therapeutic efficacy of LLL12b in suppressing disease progression in EAE in vivo and the net effects of LLL12b treatment on myelin-specific Teffs and Tregs in LLL12b-treated EAE mice. Therapeutic administration of LLL12b suppressed disease severity in 3 models of EAE, with decreased IL-17^+^ Th17 cells and increased Foxp3^+^ Tregs in LLL12b-treated mice (Figure 4), indicating that LLL12b has the capacity to suppress disease progression. As STAT3 is expressed in multiple cell types, the contribution of STAT3 inhibition in specific cell types, including CD4^+^ T cells and myeloid cells, in LLL12b-treated EAE mice needs further investigation. In addition to suppressing Th17 development and expansion, LLL12b may also suppress Th1 cells. The inhibition of STAT3 in myeloid cells impairs their antigen presenting function, which may lead to suppression of both Th1 and Th17 cells. We have previously shown that, in addition to promoting Th17 development, IL-23/STAT3 signaling promotes the encephalitogenicity of Th1 cells through STAT3/STAT4 heterodimers in Th1 cells (63), suggesting that STAT3 inhibition may also suppress Th1 cells. Further investigation that analyzes the effects of LLL12b on Th1 differentiation and encephalitogenicity in vitro and in vivo may reveal if STAT3 inhibition may suppress both encephalitogenic populations in CNS autoimmunity. To evaluate the translational potential of LLL12b, the effects of LLL12b were evaluated with PBMCs from treatment-naive MS patients. LLL12b treatment led to a Th17:Treg balance shifted toward Tregs (Figure 5). Although αCD3/CD28 was used to activate PBMCs, it is possible that LLL12b may inhibit STAT3 signaling in myeloid cells and alter the cytokine milieu, contributing to the effects we observed.

Tocilizumab, a humanized αIL-6R mAb, has been FDA approved for the treatment of several autoimmune diseases, including rheumatoid arthritis and systemic juvenile idiopathic arthritis, which demonstrates the feasibility and safety of inhibiting IL-6/STAT3 signaling systemically in human patients. Similarly, another αIL-6R mAb, satralizumab, was recently approved for the treatment of neuromyelitis optica, another human inflammatory demyelinating disease (64). However, their therapeutic potential in MS has not been characterized. αIL-6 and αIL-6R Ab have been explored in the EAE model of MS. They both are effective in preventing EAE development when given before disease onset but fail to suppress EAE progression when given after disease onset (24, 25). One of the possible reasons may be that large molecules usually have limited CNS penetration. In contrast, small molecules typically weigh less than 900 Dalton. The molecular weight of LLL12b is only 374.4 Dalton. Small molecules can penetrate the phospholipid membrane of the BBB by passive or carrier-mediated mechanisms (65) and directly exert their function on CNS-infiltrating Teffs and Tregs. Moreover, most small-molecule drugs have the advantage of being orally available compared with i.v infusion, which is required to deliver most of the large-molecule drugs. Therefore, small-molecule drug development has been the mainstay of the pharmaceutical industry. More than 90% of current on-the-market drugs are small molecules, although large molecules are quickly rising. However, multiple roadblocks have prevented the successful development of small molecules that inhibit STAT3 potently and selectively, despite decades of efforts (26). To address this challenge, we have developed a small-molecule STAT3 inhibitor LLL12, which shows great potency and selectivity, but its pharmacokinetic proprieties are not suitable for in vivo studies (Supplemental Figure 1 and Supplemental Table 1). Different approaches have been explored to overcome this limitation. Ultrasound-mediated controlled release of LLL12-loaded stimuli–responsive microdroplets has shown promising efficacy for the treatment of hypoxic cancer cells (66). As prodrug design approaches have shown great success in small-molecule drug development in the past decade, we designed and synthesized LLL12b, a prodrug of LLL12, with improved pharmacokinetic properties (Supplemental Figure 6 and Supplemental Table 3). LLL12 is enzymatically released through hydrolytic cleavage of the LLL12b carbamate group by the esterases in cells and in vivo (67). Our data suggest that the potentially novel small molecule prodrug LLL12b has promising therapeutic potential in vitro and in vivo.

More than 85% of MS patients have RRMS, and the majority of them eventually develop secondary progressive MS (SPMS), when neurologic disability continues independently of clinical relapses. More and more evidence suggests that immune dysregulation is not only a key feature of RRMS, but also progressive MS (68–75). MBP-specific Th17 and Th1 cells are higher in both primary progressive MS (PPMS) and SPMS (68, 73–75), suggesting therapeutic approaches restoring Teff:Treg balance may not only benefit RRMS, but also progressive MS. STAT3 signaling may be induced by different factors and plays important roles in multiple cell types. A phase I clinical trial of a potentially novel JAK2 inhibitor AZD1480, which led to an approximately 50% reduction of pSTAT3 in 1 patient, revealed pleiotropic neurologic adverse events (AEs), including dizziness, anxiety, ataxia, memory loss, hallucinations, and behavior changes, which were generally reversible with dose reduction or treatment cessation (76). It is unclear whether these AEs were caused by inhibition of the JAK/STAT signaling pathway or off-target effects. Further investigation is needed to evaluate whether LLL12b may have any potential pleiotropic AEs.

Reestablishing finely balanced immune responses is a key element for correcting cancer and autoimmunity, since they are the 2 opposing consequences of unbalanced immune responses. Complete and systemic blockade of STAT3 signaling in the context of a normal immune system carries the risk of increased susceptibility to infections or cancer. However, IL-6/STAT3 signaling is elevated in MS patients. Our ultimate goal is to modulate this signaling pathway through controlled administration of agents to normalize STAT3 signaling and restore Teff:Treg balance of CD4^+^ T cells of MS patients. It is worth noting that a better suppression of pSTAT3 upon LLL12b treatment appears to be associated with a relatively lower baseline STAT3 expression (Supplemental Figure 8). Future investigation of LLL12b suppression of pSTAT3 in PBMCs freshly isolated from large numbers of MS patients may help to determine the baseline pSTAT3 range that may respond well to the LLL12b treatment. Preselection of patients by their pSTAT3 levels and close monitoring of pSTAT3 levels in treated patients may be critical for achieving optimal therapeutic effects while minimizing the risk of malignancy or infection. Moreover, future investigation of novel coating and delivery systems that offer specifically targeting STAT3 in CD4^+^ T cells and/or APCs may significantly improve the benefit/risk ratio by increasing therapeutic efficacy while avoiding AEs, with the ultimate goal of treating patients with established MS.

## Methods

### Animals.

C57BL/6, B10.PL, SJL/J mice, and TCR transgenic 2D2 mice that are specific for MOG 35-55 were purchased from the Jackson Laboratory. B10.PL mice transgenic for the MBP Ac1-11–specific TCR Vα2.3 or Vβ8.2 (77) were bred in a pathogen-free animal facility at OSU. Eight- to 12-week-old male and female C57BL/6 and B10PL mice and female SJL/J mice were used for EAE studies, since male SJL/J mice are resistant to EAE induction.

### Human subjects.

Blood was obtained by leukapheresis from MS patients after informed consent. All MS patients were treatment naive for immunomodulatory drugs. Peripheral blood mononuclear cells were isolated over a Ficoll gradient and stored in liquid nitrogen until further use.

### Synthesis of LLL12b.

The synthetic route and proton NMR of LLL12b is shown in Supplemental Figures 4 and 5. The precursor LLL12 of LLL12b was prepared according to patent literature (27) with some modification. Firstly, 1-naphthalenesulfonyl chloride (compound 1; 50 g) was stirred with 28% ammonium hydroxide (300 mL) in acetone (1 L) at room temperature for about 3 hours; then, the reaction mixture was concentrated by rotary evaporation at about 60°C (water bath) to 500– 600 mL and cooled to room temperature, and 1.5 L of water was added slowly while stirring. Then, the formed white precipitate was filtered and washed with 2 L of water. After dried by air, 42 g of white powder 1-naphthalenesulfonamide (compound 2) was obtained in the yield of 91.8%. Secondly, the compound 2 (48 g) was suspended in acetic acid (480 mL) and heated to dissolve completely; it was then cooled to 40°C–45°C (water bath), and CrO_3_ (104 g) solution in H_2_O (100 mL) and acetic acid (100 mL) was added dropwise over 1–1.5 hours. The water bath temperature was maintained around 42°C. After the addition, the reaction mixture was stirred for additional 2 hours at room temperature. Then, 1 L of water was added and filtered. The obtained yellow solid was washed with large amount of water and dried by air. ^1^H NMR spectrum of the crude product (24 g) indicated that it contained about 50 % of starting material (compound 2) besides the desired 5,8-dioxo-5,8-dihydronaphthalene-1-sulfonamide (compound 3). Recrystallization from mixed solvents of acetone and hexanes can remove most starting material (compound 2). The crude product (48 g from 2 batches reaction) was dissolved in minimum acetone (about 1 L) at room temperature, and hexane (about 450 mL) was added slowly until precipitate was just observed; then, it was placed in refrigerator (about –20°C) overnight. Filtration afforded 26.9 g of compound 3 with purity of 93 %. The yield based on compound 2 was about 8.3%. Thirdly, the compound 3 (15.45 g, 65.1 mmol) was dissolved in CH_2_Cl_2_ (1.2 L) and methanol (162 mL) at room temperature and then cooled to –20°C to –15°C, and Et_3_N (1.54 mL) was added. After stirring for about 15 minutes, 3-hydroxy-1-pyrone (8.74 g, 68.75 mmol) in 300 mL of CH_2_Cl_2_ was added and stirred for about 30 minutes and then for 2–3 hours at room temperature. The formed yellow precipitate was collected by filter to get the first crop of LLL12 (3.24 g). Filtrate was concentrated at 33°C by rotary evaporation under hose vacuum to 150 mL. A total of 250 mL CH_2_Cl_2_ was added to precipitate, filtered, and washed with small quantity CH_2_Cl_2_ to get the second crop of LLL12. The total crude product was 5.27 g. Further purification by silica gel flash column chromatography was eluted with mixed solvents of acetone and hexanes (1:1, V/V) afforded 3.95 g (20% yield) of LLL12. Finally, LLL12 (2.01 mg, 6.63 mmol) was suspended in pyridine (15 g, 28.6 molecular equivalents) at room temperature; then, dimethycarbamyl chloride (1.02 g, mmol) was added and stirred at room temperature overnight. Then, the reaction mixtures were filtered and washed with CH_2_Cl_2_ and a large quantity of acetone (about 1 L) to afford the compound of LLL12b (1.08 g, yield 41 %). ^1^H NMR (in DMSO-*d*_6_, 500 MHz, 27°C) was used in the following concentrations: 8.47 (dd, ^3^*J* = 7.86 Hz, ^4^*J* = 1.01 Hz, Ar-H, 1H), 8.41 (dd, ^3^*J* = 7.81 Hz, ^4^*J* = 0.99 Hz, Ar-H, 1H), 8.09 (dd, ^3^*J* = 7.54 Hz, ^4^*J* = 0.87 Hz, Ar-H, 1H), 8.06 (t, ^3^*J* = 7.86 Hz, Ar-H, 1H), 7.96 (t, ^3^*J* = 7.95 Hz, Ar-H, 1H), 7.66 (dd, ^3^*J* = 8.05 Hz, ^4^*J* = 0.90 Hz, Ar-H, 1H), 7.45 (s, NH_2_, 2H), 3.18 (s, Me, 3H), 2.96 (s, Me, 3H).

### In vitro culture of splenocytes from TCR transgenic mice.

Splenocytes were prepared from naive 5- to 10-week-old Vα2.3/Vβ8.2 TCR transgenic mice and cultured in 24-well plates at 2 × 10^6^ cells/well with irradiated B10.PL splenocytes (6 × 10^6^ cells/well). Cells were activated with MBP Ac1-11 (10 μg/mL) and different combinations of cytokines — IL-6 (25 ng/mL), IL-23 (25 ng/mL), or TGF-β (4 ng/mL) — and LLL12b (or DMSO). For experiments in Figure 1E, splenocytes from WT/B6 mice were loaded into 24-well plates at 8 × 10^6^ cells/well with LLL12b or DMSO for 4–5 hours. The supernatant and suspension cells were then removed, and each well was washed with warm PBS 2–3 times. Purified CD4^+^ T cells from TCR transgenic 2D2 mice that are specific for MOG 35-55 were added into each well at 1 × 10^6^ to 2 × 10^6^ per well and activated with MOG 35-55 (10 μg/mL) and TGF-β/IL-6 for 3 days.

### EAE induction and in vivo treatment.

EAE was induced in 8- to 10-week-old naive C57BL/6 or female SJL/J mice by s.c. injection over 4 sites in the flank with 200 μg MOG 35-55 or PLP 139-151, respectively (C S bio) in an emulsion with CFA (DF3113605, Thermo Fisher Scientific). A total of 200 ng pertussis toxin (181 List Biological) per mouse in PBS was injected i.p. at the time of immunization and 48 hours later for C57BL/6 mice. Female SJL/J mice only received 1 injection of pertussis toxin at the time of immunization.

Splenocytes from naive 5- to 10-week-old Vα2.3/Vβ8.2 TCR transgenic mice were activated with 10 μg/mL of MBP Ac1-11 and IL-6 for 3 days. Then, the cells were washed with PBS, and 5 × 10^6^ to 10 × 10^6^ cells were injected i.p. into naive B10.PL mice.

Mice were scored on a scale of 0–6: 0, no clinical disease; 1, limp/flaccid tail; 2, moderate hind limb weakness; 3, severe hind limb weakness; 4, complete hind limb paralysis; 5, quadriplegia or premoribund state; and 6, death.

### LLL12b treatment in vivo.

LLL12b was first dissolved in DMSO. LLL12b stock solution was further diluted in a formulation of 10% DMSO/5% Tween 80 /10% PEG 400/75% PBS (Thermo Fisher Scientific). The same volume of DMSO was diluted and used as vehicle control. In total, 200 μL of diluted LLL12b or vehicle control were injected i.p. into each mouse at 10 mg/kg daily for 7 days.

### Cytokine ELISA.

ELISA was performed to detect mouse or human IL-17 in supernatant as described previously (3). Briefly, Immunolon II plates were coated with IL-17 primary Ab (2 μg/mL) (mouse, eBioscience, 14-7175-85; human, eBioscience, 14-7178-85) and incubated overnight at 4°C. The plates were then blocked with 1% BSA in PBS for 2 hours. A total of 100 μL of supernatants was added in duplicate and incubated overnight at 4°C. Then biotinylated αIL-17 secondary Ab (1 μg/mL) (mouse, eBioscience, 13-7177-85; human, eBioscience, 13-7179-85) was added and incubated for 1 hour, followed by incubation with avidin-peroxidase (2.5μg/mL) for 30 minutes. A total of 100 μL of TMB substrate was added, and stop solution was added after 5–20 minutes. Absorbance was read at 450 nm immediately. A standard curve was generated from cytokine standard, and the cytokine concentration in the samples was calculated.

### Flow cytometric analysis.

Flow cytometry staining was performed to evaluate the expression of surface markers, transcription factors (Foxp3 and RORγt), and cytokines (IL-17 and IFN-γ) in CD4^+^ T cells as described previously (3). Briefly, the cells were first incubated with Abs to the cell-surface markers for 30 minutes at 4°C, followed by treatment with Cytofix/Cytoperm solution from BD Bioscience (catalog 554714) (for IL-17) for 20 minutes or from eBioscience (Thermo Fisher Scientific, 00-5523-00) (for Foxp3 and RORγt) for 1 hour at 4°C. Then, cells were stained for intracellular IL-17 or transcription factors (Foxp3 and RORγt) for 30 minutes. Phospho flow cytometry was used to detect pSTAT3 in CD4^+^ T cells. The cells were fixed using BD Cytofix Fixation buffer for 10 minutes at 37°C and permeabilized using ice-cold Perm buffer III (BD Biosciences, 558050) for 30 minutes at 4°C. Then, the cells were stained for surface markers and pSTAT3 for 1 hour. Approximately 100,000 live cell events were acquired on a FACSCantoII and analyzed using FlowJo software (Tree Star Inc.). Fixable viability dye eFluor 780 was used to determine live cell population. PE anti–mIL-17 (catalog 506904), FITC anti-mCD4 (catalog 100406), AF647 anti-Foxp3 (catalog 320014), Pacific Blue anti-CD44 (catalog 103020), PE-Cy7 anti-mCD25 (catalog 102016), PE anti–hIL-17 (catalog 512306), FITC anti-hCD25 (catalog 302604), and PCP anti-hCD45RA (catalog 304156) were purchased from BioLegend. AF647 anti-STAT3 (pY705) (catalog 557815) was purchased from BD Biosciences. PE anti-RORγt (catalog 50-112-9700) and fixable viability dye eFluor 780 (catalog 65-0865-18) were purchased from Thermo Fisher Scientific.

### CFSE-based suppression assay.

For experiments in Figure 3, C and D, CD4^+^ T cells from WT/B6 mice were activated on 24-well plates with plate-bound anti-CD3/CD28 (1 μg/mL) plus TGF-β (4 ng/mL) and IL-6 (25 ng/mL) for 3 days, in the presence of LLL12b (0.25 μM) or DMSO. Meanwhile, splenocytes from naive TCR transgenic 2D2 mice that are specific for MOG 35-55 were labeled using CellTraceTM CFSE Cell Proliferation Kit (Thermo Fisher Scientific). The iTreg cultured cells were washed and mixed with the CSFE-labeled splenocytes from 2D2 mice at 1:4 ratio in the presence of 10 μg/mL MOG 35-55 peptide for 4–5 days. The CFSE-labeled CD4^+^ Teffs were evaluated using flow cytometry. For experiments in Figure 5M, iTregs were generated by culturing PBMCs from MS patients on 24-well plates with plate-bound anti-CD3/CD28 (1 μg/mL) plus TGF-β1 (5 ng/mL), IL-2 (500 U/mL), and all trans retinoic acid (10 nM) for 72–96 hours. Autologous PBMCs from the same MS patient were labeled using the CellTraceTM CFSE Cell Proliferation Kit (Thermo Fisher Scientific) by adding CFSE to a final concentration of 0.5–5 μM, followed by a 15-minute incubation at 37°C in the dark. The CFSE-labeled PBMCs were then cultured with LLL12b (or DMSO) (0.25 μM) for 2 hours, washed with warm PBS, and mixed with iTreg cultured cells from the same patient (Teff:Treg = 16:1) in the presence of anti-CD3/CD28 for 4–5 days. CFSE in CD4^+^ T cells was determined by flow cytometry. The percentage of suppression of LLL12b or DMSO-treated CD4^+^ T cells by Tregs from the same patient was calculated. The percentage of suppression = 100 – (% proliferating CFSE^+^ cells in each ratio/% proliferating CFSE^+^ cells without Tregs) × 100.

### Data and materials availability.

All data associated with this study are included in this paper or the supplementary materials. A patent has been filed for the compound LLL12b in autoimmune diseases. LLL12b may be obtained through a material transfer agreement (MTA) with OSU.

### Statistics.

A statistically significant difference in EAE clinical scores was considered to be *P* < 0.05, as determined by Mann-Whitney *U* test. ELISA and quantitated flow data comparisons were performed using 2-tailed unpaired Student’s *t* test with 2 groups and 1-way ANOVA with 3 or more groups. The percentage of suppression in Figure 3D was compared using Mann-Whitney *U* test. The Wilcoxon matched-pairs signed rank test or paired 2-tailed Student’s *t* test was used to compare the difference between each set of matched pairs of human data in Figure 5.

### Study approval.

All animal protocols were approved by the OSU IACUC. The study using human PBMCs of MS patients was performed under OSU IRB protocol no. 2015H0076 with written informed consent received from participants prior to inclusion in the study.

## Author contributions

YY conceptualized and designed research, analyzed the results, and wrote the manuscript. SIA, EEK, MFF, and EYZ performed the experiments. WP provided support of mouse studies. CL conceptualized the STAT3 small molecule inhibitor prodrugs design, mapped the structural design, and designed the synthetic routes. XY and JS prepared the experimental agents. AELR and MKR collected PBMCs from treatment-naive MS patients and helped with data discussion and manuscript review. All authors read and approved the final manuscript.

## Supplementary Material

Supplemental data

## Figures and Tables

**Table 1:**
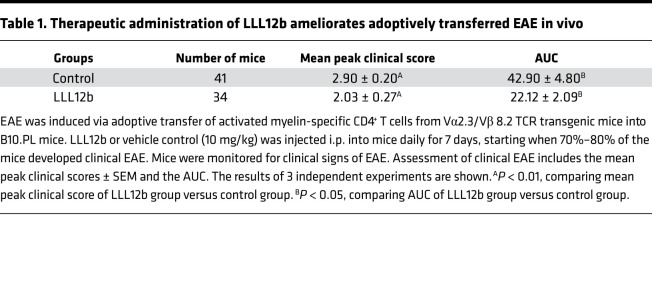
Therapeutic administration of LLL12b ameliorates adoptively transferred EAE in vivo

**Table 2:**
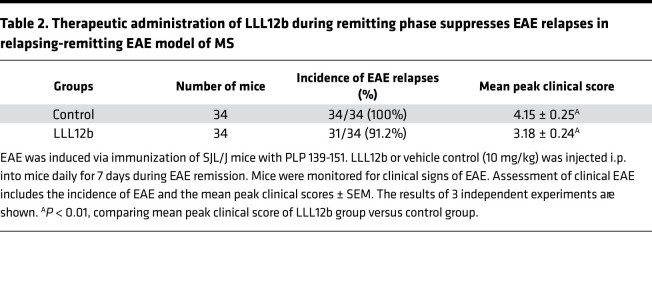
Therapeutic administration of LLL12b during remitting phase suppresses EAE relapses in relapsing-remitting EAE model of MS

## Version 1 01/07/2021

In-Press Preview

## Version 2 02/22/2021

Electronic publication
