# Supplementary material for: A STAT3 inhibitor ameliorates CNS autoimmunity by restoring Teff:Treg balance
Source: JCI Insight. 2021 Feb 22;6(4):e142376. doi: 10.1172/jci.insight.142376 (PMC7934926; doi:10.1172/jci.insight.142376)
Supplement: Supplemental data [file jciinsight-6-142376-s123.pdf]

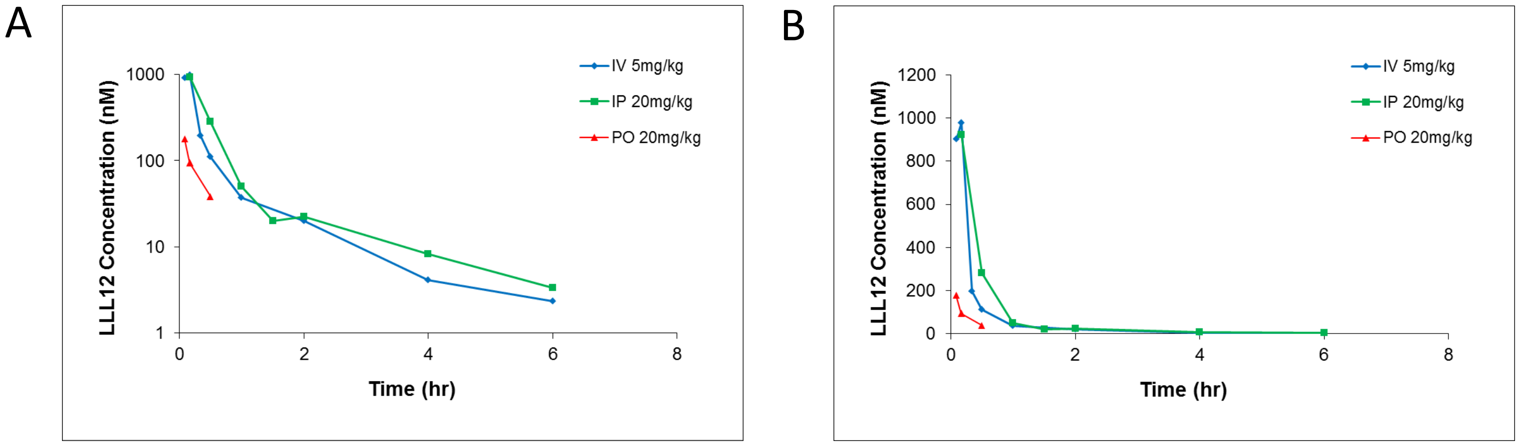

Figure S1. Overlaid semi logarithmic (A) and linear (B) plots of concentration-time profiles of LLL12 in mice by IV, IP and oral routes. The IP 5min time point was removed.

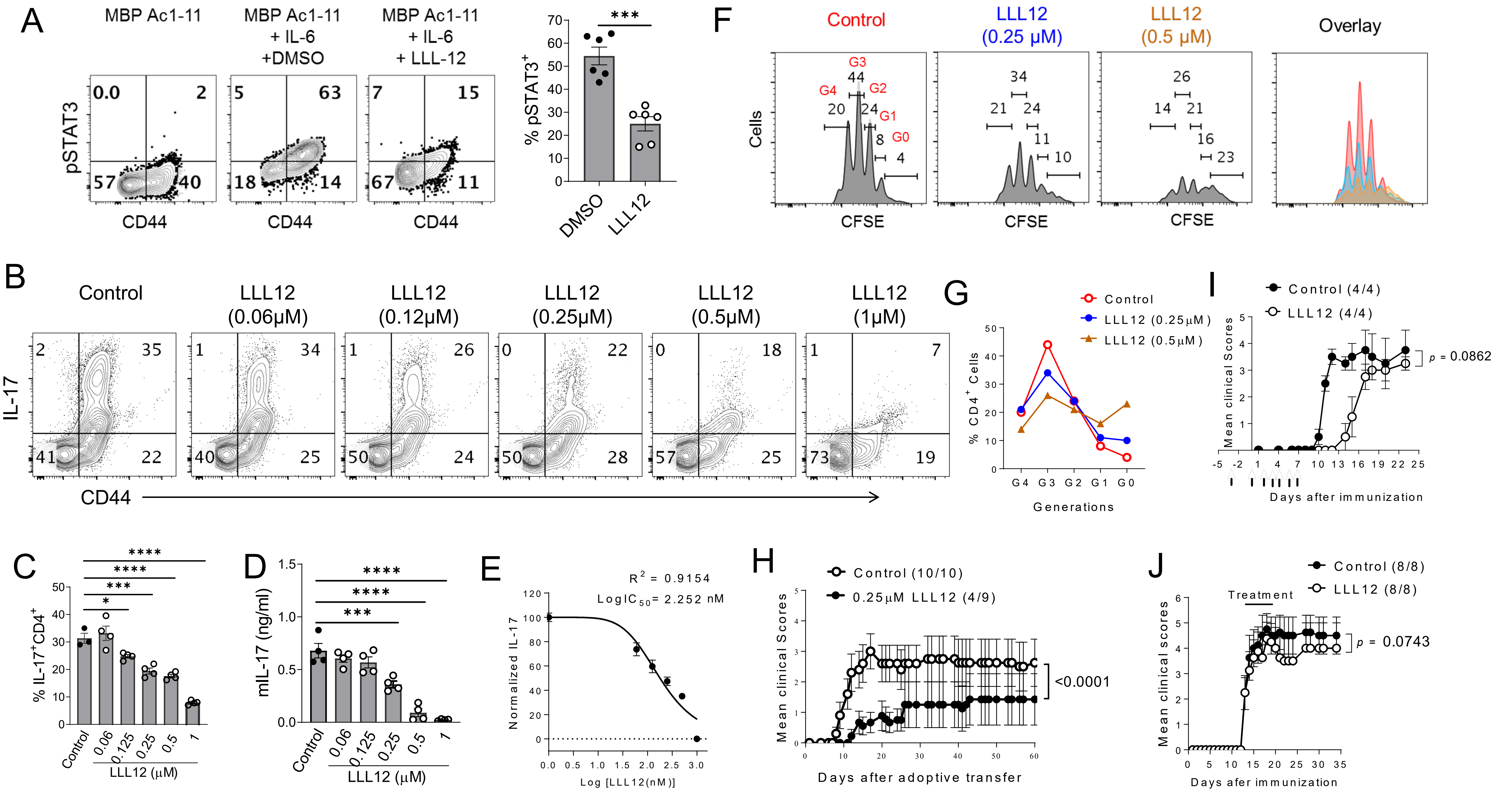

Figure S2. Small molecule STAT3 inhibitor LLL12 suppresses Th17 development. (A) Splenocytes from naïve Vα2.3/Vβ8.2 TCR transgenic mice that are specific for MBP Ac1-11 were cultured with IL-6 for 30' in the presence of LLL12 (0.25 μM) or DMSO (vehicle control). pSTAT3 was determined by phospho flow cytometry. Cells were gated on $CD4^+$ cells. Group means (n=3) were compared with unpaired Student t-test. (B-E) Splenocytes from naïve Vα2.3/Vβ8.2 TCR transgenic mice were activated with MBP Ac1-11 plus TGFβ1 and IL-6 for 3 days in the presence of different concentrations of LLL12 or DMSO. (B) IL-17 in CD4 T cells was determined by intracellular flow cytometric analysis. Cells were gated on $CD4^+$ T cells. (C) Mean percentage of $IL\text{-}17^+CD4$ T cells of LLL12-treated groups were compared to DMSO group by one-way ANOVA (n=3). (D) IL-17 in supernatant was determined by ELISA. Mean IL-17 of LLL12-treated groups were compared to the DMSO group with one-way ANOVA (n=4). (E) Dose response curve of LLL12 concentration and normalized inhibition of the percentage of $IL\text{-}17^+$ CD4 T cells. (F-G) Splenocytes from naïve Vα2.3/Vβ8.2 TCR transgenic mice were labeled with CFSE and activated with MBP Ac1-11 and IL-6 for 5 days, in the presence of different concentrations of LLL12 or DMSO. (F) CFSE was determined by flow cytometric analysis. Cells were gated on $CD4^+$ T cells. The amount of cells per generation in (F) was summarized in (G). (H) Splenocytes from naïve Vα2.3/Vβ8.2 TCR transgenic mice were activated with MBP Ac1-11 and IL-6 for 3 days in the presence of LLL12 (0.25 μM) or DMSO. The cells were then collected, washed and injected ip into naïve B10PL recipient mice. (I-J) Naïve C57BL/6 mice were immunized with MOG 35-55. The mice were injected ip with LLL12 (10mg/kg) or vehicle control for 7 days starting either day -2-4 (I) or day 10-16 (J) after immunization. (H-J) EAE was monitored. Disease incidence (sick mice/total mice) is indicated in parentheses. A statistically significant difference was considered to be $P<0.05$, as determined by Mann-Whitney U test. All error bars denote s.e.m. * denotes $p<0.05$; * * denotes $p<0.01$; *** denotes $p<0.001$; **** denotes $p<0.0001$.

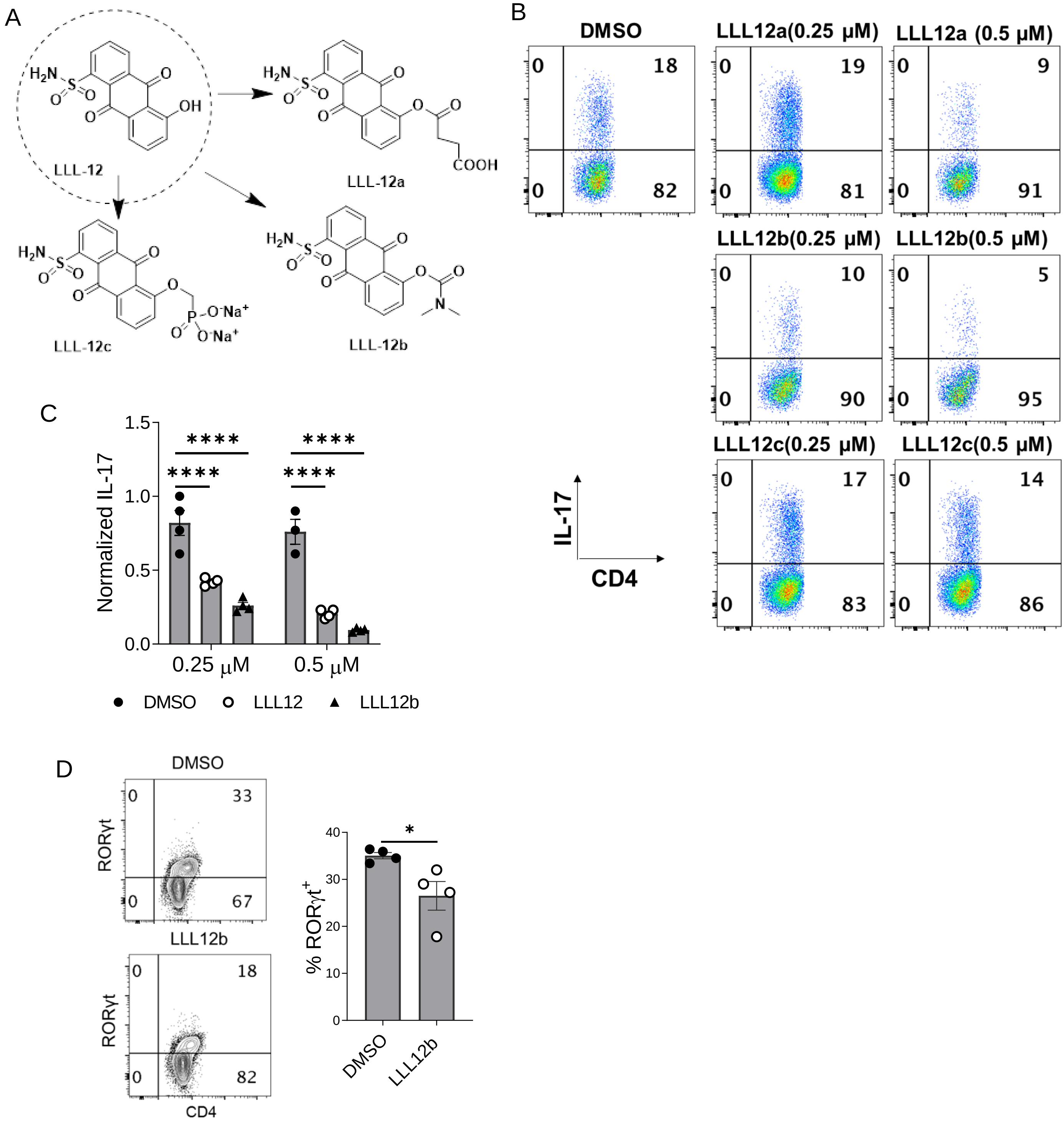

Figure S3. Novel LLL12 prodrugs suppress IL-17 production in myelin-specific CD4 T cells. (A) Structure of novel LLL12 prodrugs. (B-D) Splenocytes from naïve Vα2.3/Vβ8.2 TCR transgenic mice were activated with MBP Ac1-11 plus TGFβ and IL-6 for 3 days in the presence of different concentrations of LLL12, LLL12 prodrugs or vehicle control DMSO. IL-17 in CD4 T cells was determined by intracellular staining. Cells were gated on $CD4^+$ T cells (B). IL-17 in supernatant was analyzed by ELISA and compared with two-way ANOVA (n=4) (C). RORγt in CD4 T cells in LLL12b and control groups was determined by intracellular staining, gating on CD4 T cells, and compared with unpaired student T-test (D). Data represent three independent experiments.

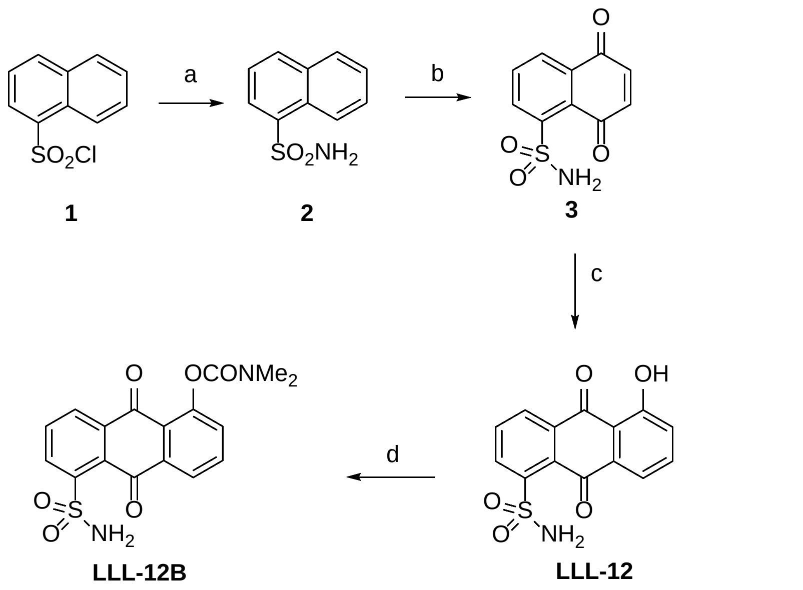

a: acetone, $NH_3 \cdot H_2O$, rt, 3h; b: Cr_2O_3, $HOAc/H_2O$, water bath tempture 40~45 ^oC;
c: $CH_2Cl_2/MeOH$, Et_3N (0.02 eq), 3-hydroxy-1-pyrone, -20 ~ -10^oC,then rt 2-3h;
d: pyridine (28.6 eq), rt, $ClCONMe_2$

Figure S4. The synesthetic route of LLL12b.

Figure S5. Proton NMR of LLL12b.

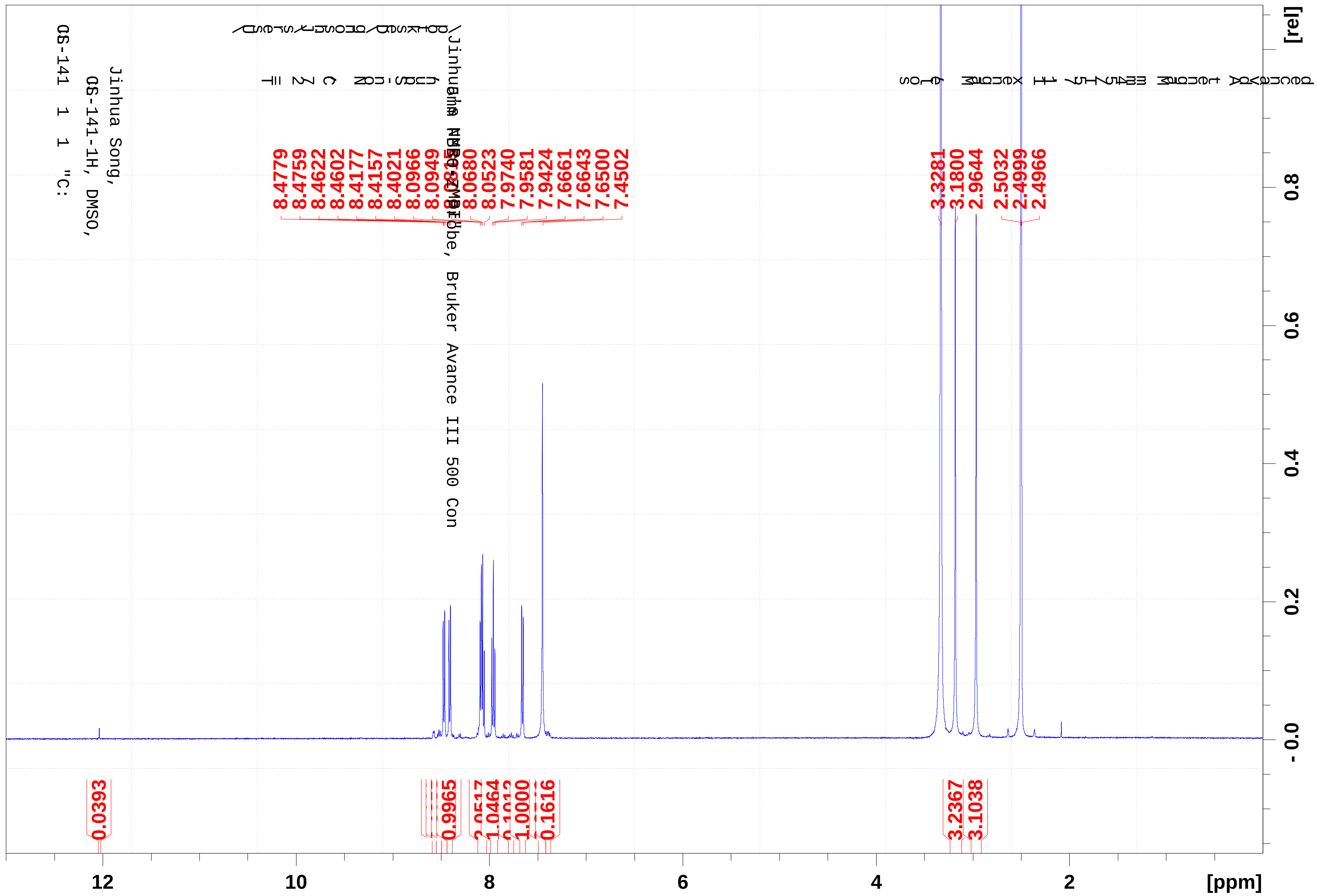

CL-JS-141 1 1 "C:\Users\jhsong\Desktop\Jinhua's NMR---MBI"

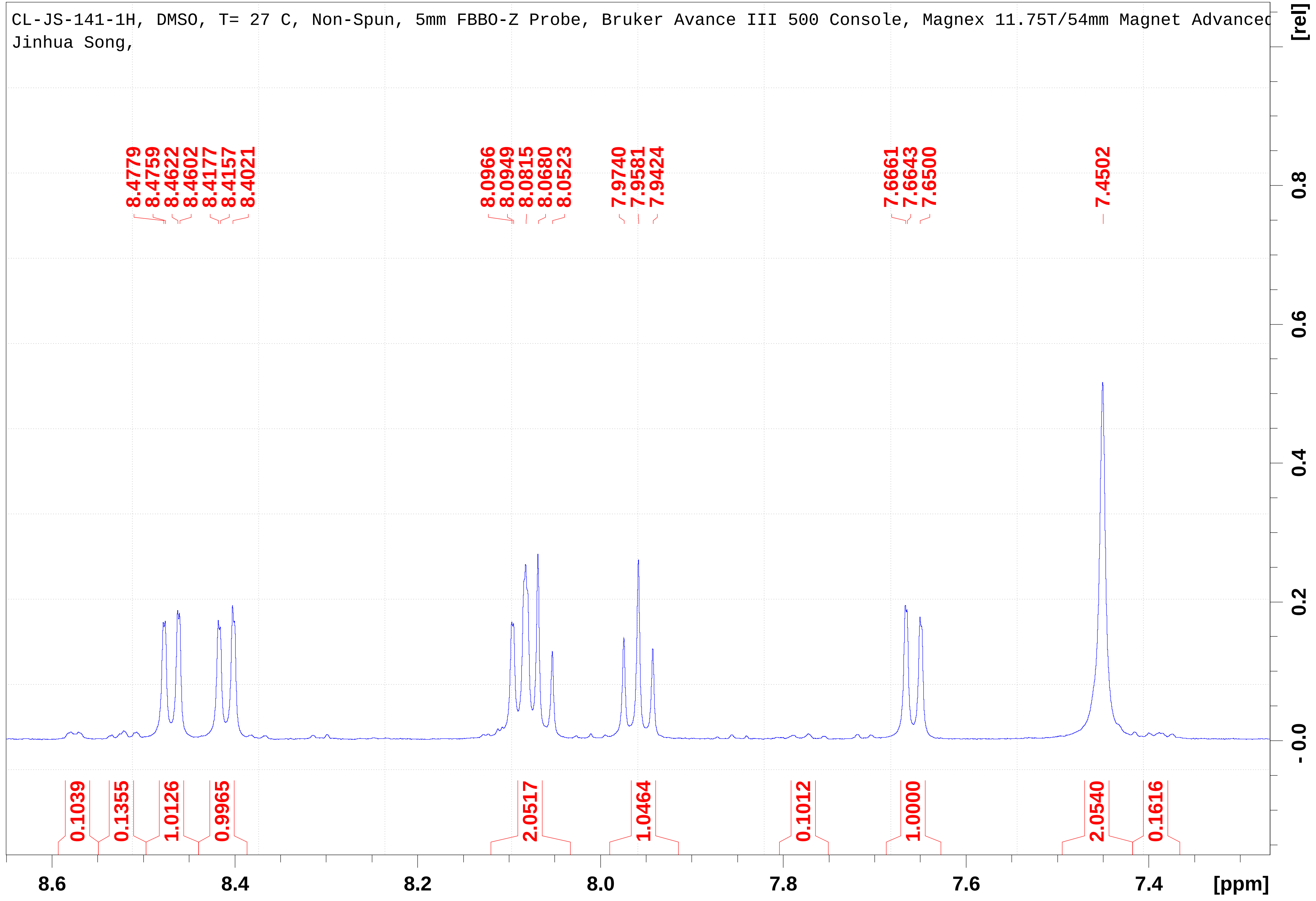

CL-JS-141 1 1 "C:\Users\jhsong\Desktop\Jinhua's NMR---MBI"

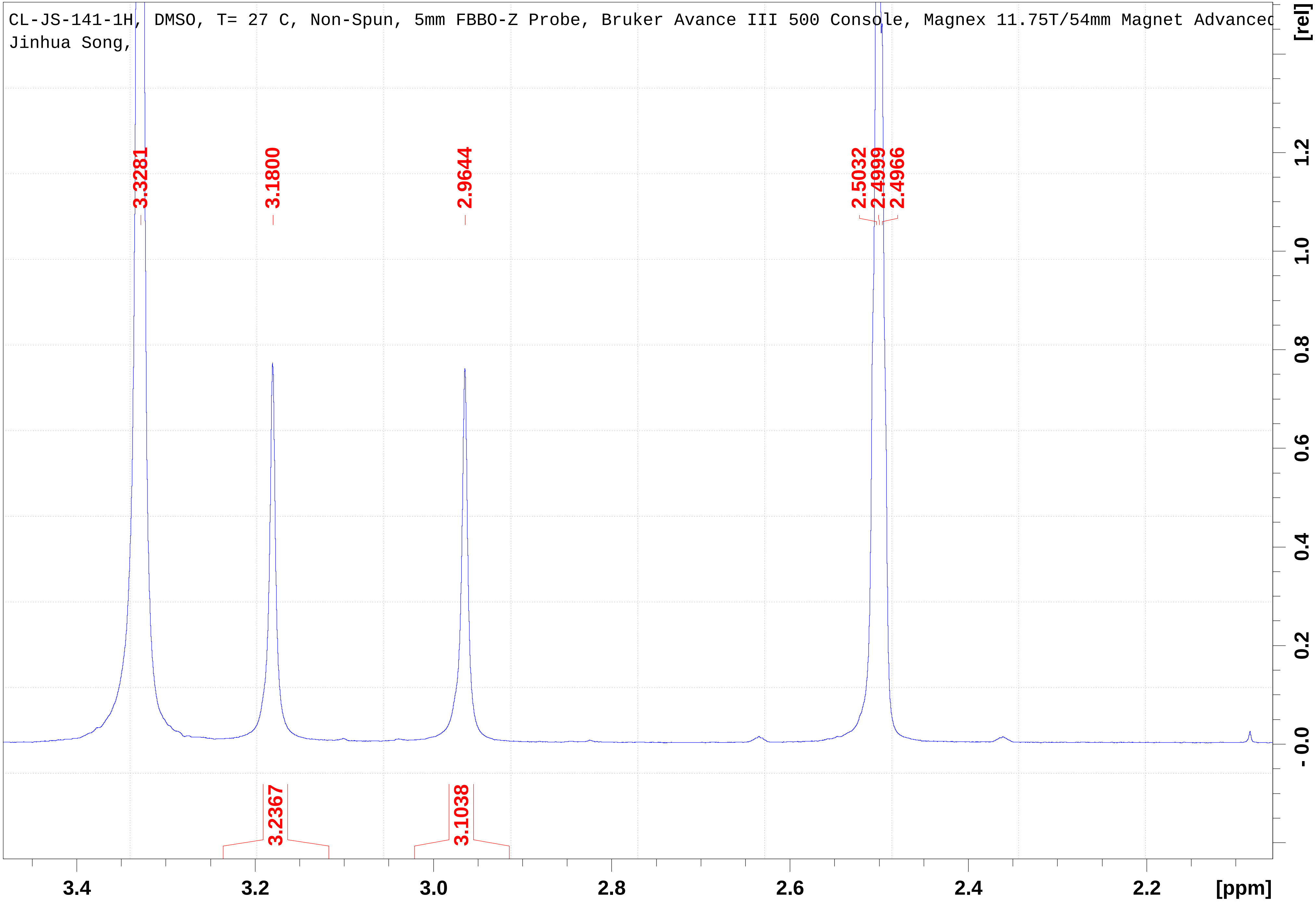

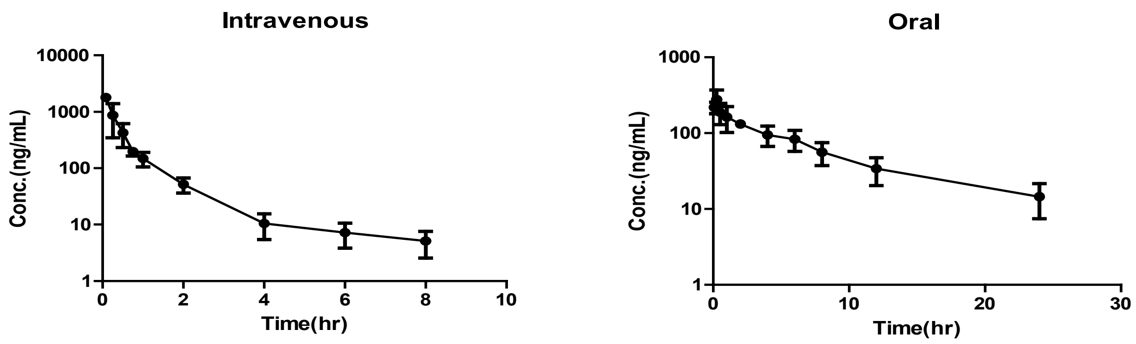

Figure S6. Linear plots of concentration-time profiles of LLL12b in mice by IV and oral routes.

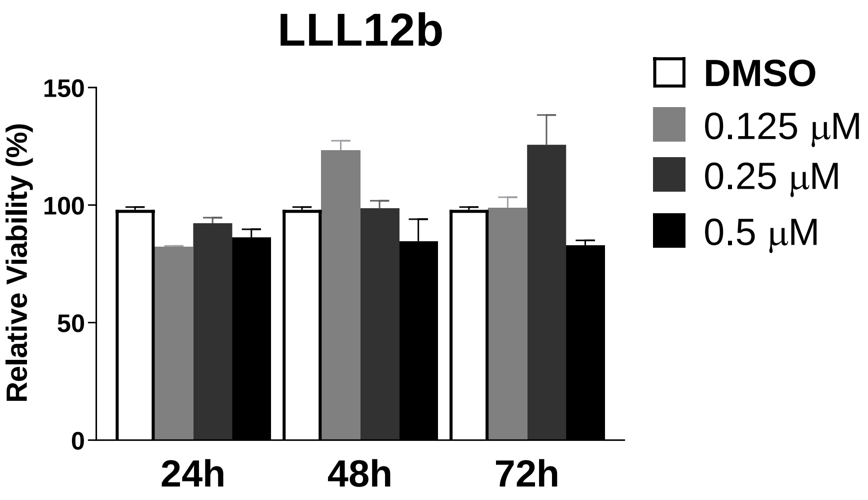

Figure S7. Trypan blue exclusion test. Splenocytes from naïve TCR transgenic mice that are specific for MBP Ac1-11 were cultured with MBP Ac1-11 plus different concentrations of LLL12b for 3 days. DMSO was used as a vehicle control. Viability (the percentage of viable white cells) was calculated as by dividing the number of viable white cells by the number of total cells (blue and white cells) in each condition. Relative viability was calculated by normalizing the viability of each condition to the viability of the group cultured with DMSO.

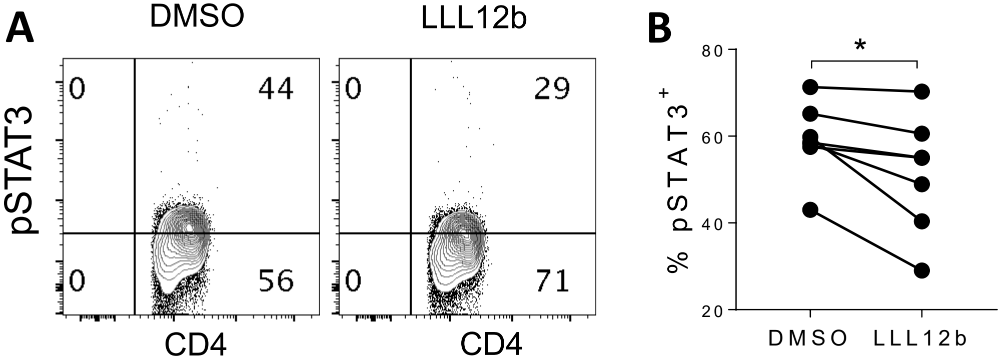

Figure S8. LLL12b suppresses IL-6-induced STAT3 phosphorylation in human CD4 T cells from MS patients. PBMCs from treatment-naïve MS patients were activated with αhCD3 plus IL-6 for 30 minutes in the presence of DMSO or LLL12b (0.25 μM). (A) pSTAT3 was determined by phospho flow cytometry. Cells were gated on $CD4^+$ cells. (B) pSTAT3 in LLL12b and DMSO treated groups from treatment-naïve MS patients (n=6) were compared with Wilcoxon matched-pairs signed rank test for significance ($P<0.05$). * denotes $P<0.05$.

Table S1 The area-under-curve and relative bioavailability of LLL12 in mice dosed IV, IP and PO

PK parameters	Unit ↓ (Dosage→)	LLL12		
		IV	PO	IP
		5mg/kg	20mg/kg	20mg/kg
Tmax	hr	0.167	0.083	0.167
Cmax	nM	978	176	922
AUCall	nM*hr	373	40.7	431
AUC inf_obs	nM*hr	377	NA	438
F	%	100	2.73	29.1

Table S2. EAE development in B10PL mice that were adoptively transferred with splenocytes from Vα2.3/Vβ 8.2 TCR transgenic mice that were activated with MBP Ac1-11 plus LLL12 or vehicle control *in vitro*.

Groups	Number of mice	Incidence of EAE (%)	Mean area under the curve	Mean day of onset of EAE mice	Mean peak clinical score of EAE mice	Mean peak clinical score of all mice
Control	20	19/20 (95%)	107.6 ± 22.85	8.6 ± 0.68	4.1 ± 0.45	3.85 ± 0.47[a]
LLL12	19	12/19 (63%)	52.47 ± 4.12	9.7 ± 0.85	3.5 ± 0.54	2.21 ± 0.52 [a]

[a] Mean peak clinical score of all mice: Control vs LLL12b (p<0.05)

Table S3. The area-under-curve and relative bioavailability of LLL12b in mice dosed IV and PO

Parameters	**Intravenous**	**Oral**
	**2 mg/kg**	**8 mg/kg**
**Co/Cmax (ng /mL)**	2879.87 ± 934.57	277.75 ± 92.96
**Tmax (hr)**	-	0.19 ± 0.09
**$AUC_{0\text{-}t}$(hr*ng / mL)**	892.29 ± 130.87	1356.77 ± 395.87
**t1/2 (hr)**	3.88 ± 0.45	7.68 ± 1.91
**Vd (L / kg)**	12.43 ± 2.89	
**Cl (L / hr/ kg)**	2.19 ± 0.27	
**F (%)**	**38.01%**	
